# Intact Dendritic Cell Pathogen-Recognition Receptor Functions Associate with Chronic Hepatitis C Treatment-Induced Viral Clearance

**DOI:** 10.1371/journal.pone.0102605

**Published:** 2014-07-17

**Authors:** Ian Gaël Rodrigue-Gervais, Hawley Rigsby, Loubna Jouan, Bernard Willems, Daniel Lamarre

**Affiliations:** 1 Centre de Recherche du Centre Hospitalier de l'Université de Montréal (CR-CHUM), Montréal, Québec, Canada; 2 Département de Médecine, Université de Montréal, Montréal, Québec, Canada; McMaster University, Canada

## Abstract

Although studies have addressed the exhaustion of the host's immune response to HCV and its role in treatment, there is little information about the possible contribution of innate immunity to treatment-induced clearance. We hypothesized that because intact myeloid dendritic cell (MDC) pathogen sensing functions are associated with improved HCV-specific CD8^+^ T cell functionality in some chronically infected patients, it might enhance HCV clearance rate under standard interferon therapy. To investigate this hypothesis, TLR-induced MDC activation and HCV-specific CD8^+^ T cell response quality were monitored longitudinally at the single-cell level using polychromatic flow cytometry in chronically infected patients undergoing interferon therapy. We correlated the immunological, biochemical and virological data with response to treatment. We demonstrate that the clinical efficacy of interferon-induced viral clearance is influenced by the extent to which HCV inhibits MDC functions before treatment, rather than solely on a breakdown of the extrinsic T cell immunosuppressive environment. Thus, viral inhibition of MDC functions before treatment emerges as a co-determining factor in the clinical efficacy of interferon therapy during chronic HCV infection.

## Introduction

An estimated 185 million people are persistently infected with the hepatitis C virus (HCV) [1]. HCV infection causes a progressive liver disease that leads to cirrhosis and cancer. People can be infected by one of several genetic types of HCV, each of which varies in its sensitivity to therapy; genotype 1 HCV being the most difficult to treat [2]. Prior to the advent of direct antiviral agents (DAA), the only approved treatment for HCV infection was weekly injection of polyethylene glycol-conjugated interferon-a2a or -a2b (pegIFN) combined to daily oral ribavirin. This 48-week IFN treatment course is often poorly tolerated and achieves a sustained virological response (SVR) in only 55% of infected patients. Direct-acting antiviral molecules targeting HCV's serine NS3/4A protease are now approved for use in combination with pegIFN for the treatment of HCV [3]. However, emergence of drug-resistant viruses remains a challenging hurdle [4] and not all patients respond to DAA [5]. Alternative combinatory treatment strategies are therefore still needed.

Generally the natural immune response to HCV is not effective at eradicating the virus. Nonetheless, polyfunctional HCV-specific T cell responses (characterized by coordinate cytotoxic degranulation and IFN-γ, IL-2 and TNFα expression) have been associated with control of viremia in spontaneous resolution and upon antiviral intervention during or shortly after acute infection (reviewed in [6]). Dendritic cells (DCs) are essential for initiating and maintaining virus-specific cellular immunity to chronic infections [7]. Recent studies have shown that the adoptive transfer of autologous DCs loaded with chemically inactivated HIV-1 or SIV led to considerable viral suppression in therapy-naïve chronically infected humans and macaques [8], [9]. But the durability of control with this approach is uncertain in the setting of chronic HCV infection [10] given the complex interplays of suppressive pathways that underlie the exhaustion of T cells. DCs may not be enough, and in some cases, may not even be strictly necessary during antiviral therapy. A therapeutic vaccine may help amplify pre-existing T cell responses that are not protective, or at least not sufficiently protective [11]. A more effective approach in such instances might be to target the exhausted T cells directly in order to retool them to be more immunoprotective. This is the goal of anti-programmed death (PD)-1-based therapies that seek the breakdown of the immunosuppressive environment. While early pre-clinical studies have indicated that treatment with anti-PD1 exerts some beneficial effect in HCV infection [12], conceivably, harnessing the natural APC functions of DCs might also contribute to reprogramming T cells to help clear the infection.

Intact innate functions of myeloid DCs (MDCs) at the level of pathogen sensing by TRIF-scaffolded TLRs has been linked to the maintenance of polyfunctional HCV-specific effector CD8^+^ T cells under circumstances of inhibitory PD-1 receptor signaling in chronically HCV-infected patients [13]. HCV inhibits TLR3-mediated antiviral responses through NS3/4A proteolytic cleavage of its adaptor molecule TRIF within infected cells [14], [15] and it would appear that HCV inhibits these very functions in MDCs [16], [17]. Because TLR signaling has been reported to lessen T cell exhaustion in persistent viral infections [18] and DAAs can restore select TLR signaling of HCV inhibited MDCs *in vitro* [13], we hypothesized that the HCV clearance rate under pegIFN/ribavirin therapy could be enhanced in those patients with intact MDC pathogen sensing functions. Here we present evidence that the extent to which HCV inhibits TRIF-dependent pathogen recognition functions of MDCs before treatment is a co-determining factor in the clinical efficacy of pegIFN antiviral therapeutic modalities in chronic HCV infection.

## Materials and Methods

### Study participants

Patients with chronic HCV infection initiating a 48-wk course of pegIFN and ribavirin treatment were enrolled into a longitudinal study by the hepatology experimental unit of Hôpital Saint-Luc. All procedures were in accordance with our institutional ethical commission and with the Helsinki Declaration. This study was approved by the ethical commission of the CHUM, Hôpital St-Luc, Montréal, Canada (SL05.025). All subjects gave written informed consent prior to study entry. Thirty-four patients with at least one visit before therapy were randomly selected from this cohort for this study (Table 1). Twenty of the 34 selected participants had multiple longitudinal PBMC visits during and after treatment (Table 2 lists the clinical characteristics of these patients). Blood was collected in heparin preparation tubes (Vacutainer Systems, BD Biosciences, Franklin Lakes, NJ) at 0 (pre-treatment), 4, 8, 12, 24 and 48 wks during therapy and at 12 and 24 weeks post-treatment. PBMCs were obtained by standard Ficoll-Paque density gradient centrifugation and cryopreserved at −140°C at 10x10^6^ cells/ml in FCS (Sigma-Aldrich, St. Louis, MO) containing 10% DMSO until use. All samples used in this study were from patients documented to be HCV-seropositive for >1 yr, hepatitis B virus- and HIV-1-seronegative, not receiving antiviral therapy for >6 months before treatment initiation and to have a plasma viral RNA load (PVL) of >10^5^ IU/ml. Treatment response and non-response (NR) were defined according to standard definitions [19]. Subjects with insufficient viral response at 12 or 24 wks discontinued therapy per protocol as treatment failures. In order to ensure that only non-responders with adequate drug exposure were evaluated (true biological non-responders), we included only patients with a minimum of 12 wks of therapy and compliance of greater than 80%. Patients were classified into either the cytokine profile-defective (CP-D) versus cytokine profile-normal (CP-N) MDC function group for LPS and polyinosinic:polycytidylic acid [poly(I:C)] stimulatory conditions at study entry (see *FACS analysis* and Table 1) before any knowledge of treatment outcome.

**Table 1:** Clinical data of the 34 patients clustered according to MDC functionality suffering from chronic HCV and undergoing pegIFN treatment.

		CP-N	CP-D
Patients (%)		21 (62)	13 (38)
Age^1^		46±10	49±6
% male		67	69
Genotype (%)	HCV1a/b	15 (71)	12 (92)
	HCV2a/b/c	4 (19)	
	HCV3/3a	2 (10)	1 (8)
Pre-treatment PVL^2^		6.24±0.72	6.26±0.57
SVR (%)	All genotypes	12 (57)	1 (8)
	HCV1a/b	6 (40)	1 (8)
	HCV2a/b/c	4 (100)	NA
	HCV3/3a	2 (100)	NA

In parentheses are %; NA, not applicable.

1 yr ± SD.

2 log_10_ IU x ml^−1^ ± SD.

**Table 2:** Characteristics of the twenty HCV chronic patients longitudinally followed during treatment course.

Patient ID	Age/Sex	HCV Genotype	Plasma HCV^a^	ALT (UxL^-1^)	AST (Ux L^-1^)	MDC cluster group	PBMC Viability^b^	CD8^+^ Ag-specifc T cells			Therapy Outcome
								HCV		CEF	
								%^c^	Proteins^d^	%^c^	
P47	50/M	2b	6.35	126	60	CP-N	≥95%	ND	ND	ND	SVR
P55	54/M	1a	6.07	130	72	CP-N	≥95%	0.32	3	0.05	SVR
P64	52/M	1a	6.87	48	37	CP-N	≥95%	0.01	1	0.65	NR
P69	45/M	1a	6.53	88	40	CP-N	≥95%	0.32	3	0.23	SVR
P70	49/F	1a	6.43	40	37	CP-N	≥95%	2.04	2	2.04	SVR
P71	45/F	1a	5.29	62	54	CP-N	≥95%	0.01	1	1.54	NR
P74	50/F	1a	7.13	22	23	CP-N	low	ND	ND	ND	SVR
P75	P75	1a	6.60	52	48	CP-N	low	ND	ND	ND	SVR
P76	68/M	1a/b	6.92	43	45	CP-N	≥95%	0.73	4	0.67	NR
P77	43/M	1	6.16	222	115	CP-N	low	ND	ND	ND	SVR
P78	52/M	2	6.46	186	160	CP-N	low	ND	ND	ND	SVR
P79	33/F	2	6.53	49	33	CP-N	≥95%	ND	ND	ND	SVR
P80	51/F	2b	6.05	117	80	CP-N	≥95%	ND	ND	ND	SVR
P82	45/M	3	4.01	81	40	CP-N	low	ND	ND	ND	SVR
P83	44/M	3a	5.18	39	27	CP-N	≥95%	ND	ND	ND	SVR
											
P48	43/M	1a	5.85	42	34	CP-D	≥95%	0.01	1	0.15	NR
P49	60/F	1b	5.82	91	80	CP-D	≥95%	0.03	1	0.31	NR
P60	49/M	1a	6.23	178	71	CP-D	≥95%	0.01	2	0.03	SVR
P65	44/F	1b	6.16	43	60	CP-D	≥95%	0.08	1	0.36	NR
P84	43/M	3a	5.81	172	134	CP-D	≥95%	ND	ND	ND	NR

a log_10_ IU/ml.

b By trypan blue count; visits during and following treatment.

c Frequencies are total cytokine-producing (IFN-γ, IL-2, TNFα) and degranulating (CD107a^+^) cells out of the CD8^+^ memory subset at pre-treatment. All positive responses to HCV pools were summed to determine the total antigen-specific response within the memory peripheral blood T cell populations. Due to HCV peptide reagent availability, only genotype 1 patients could be stimulated. ND: not determined.

d Number of HCV proteins detected by CD8^+^ T cells.

### TLR agonists and peptides

The following TLR agonists were used at the indicated final titrated concentrations: poly(I:C) (TLR-3 agonist; 25 µg/ml) and ssRNA40 (TLR-7/8 agonist; 5 µg/ml) from InvivoGen (San Diego, CA); LPS (*Escherichia coli* 055:B5, TLR-4 agonist; 0.1 µg/ml) from Sigma-Aldrich; 3M-002 (TLR-8 agonist; 5 µM) and 3M-011 (TLR-7/8 agonist; 5 µM) both provided by 3M Pharmaceuticals (St Paul, MN). Peptides (18-mers overlapping by 11) corresponding to HCV H77c genotype 1 strain full-length Core, NS3, NS4A/B, and NS5B (National Institutes of Health AIDS Research and Reference Reagent Program) were used at 2 µg/ml each. Six peptide mixes (0.2 mg/ml per peptide) were prepared: one each for full-length Core and NS4A/B and two each for full-length NS3 and NS5B. Each HCV pool consisted of 45–50 peptides. A pool of 32 MHC class I-restricted 9-mer peptides covering immunodominant regions of CMV, EBV, and influenza (CEF) were purchased from PANATecs (Tübingen, Germany) and used as described above.

### Viral load assays

Plasma HCV RNA was measured using the Cobas AmpliPrep/TaqMan HCV Test (Roche Diagnostics, Branchburg, NJ) at the Laboratoire de Santé Publique du Québec.

### Flow cytometry assays

Thawed PBMC samples were examined for viability by trypan blue exclusion after a rest period of 12 h at 37°C in the presence of DNAse (10 U/ml) and excluded from further analysis if viability was below 95%. CD107, IL-2, IFNγ- and TNFα-expressing T cells as well as IL-12, IL-6 and TNFα-expressing MDC, following *ex vivo* stimulation with peptide pools or TLR agonists respectively, were detected by previously described assays [13], [16]. We determined cell viability by ViViD dye staining. Data were acquired on an LSRII (BD Biosciences) equipped for the detection of 11 fluorescent parameters. A minimum of 1.0x10^6^ and 1.25x10^6^ total PBMCs was collected by FACS for each MDC and Ag-specific T cell sample, respectively. Data analysis was performed using FACS DiVa version 6.0 software. Cytokine expression profile (CP) unsupervised hierarchical clustering analyses of patients into inhibited (CP-D) and intact (CP-N) MDC pathogen sensing function were done as described previously [16] using the log_2_ ratio of geometric mean fluorescence intensity (MFI^stimulated^/MFI^unstimulated^) values of MDCs positive for IL-12 and TNFα for LPS- and poly(I:C)-stimulatory conditions at study entry (referred to as week 0, pre-treatment). The CP clustering analysis was blinded to treatment outcome. For these unsupervised clustering analyses in TMeV array software (version 4.9; http://www.tm4.org/mev.html), protein expression in lineage^−^CD16^−^CD45^+^CD11c^+^MHC-II^br^ MDCs from HCV patients treated with stimuli was presented as the log_2_ change versus protein expression in similarly stimulated MDCs from age-matched aviremic control donors (n = 12). Boolean combinations of single-function gates were created to determine the frequency of each Ag-specific T cell response based on all possible combinations (up to 15) of cytokines and CD107a expression. A lower threshold, corresponding to two SDs above healthy donor background, was built for each specific functional combination, and values below this threshold were set to zero. Total frequencies of virus-specific CD8^+^ T cells were calculated by summing the frequencies within each unique combination of functions (counting each responding cell only once) and were normalized to the memory subset (defined as CD3^+^CD4^−^CD8^+^ cells that were not CD27^+^CD45RO^−^). Because HCV proteins were analyzed in parts, a positive response by any peptide submix constituted an HCV-specific response, and if one or more such positive responses were identified, these responses were summed to calculate a total Ag-specific response frequency within the memory peripheral blood T cell population.

### Real-time qPCR

Total cellular RNA from PBMCs was extracted using the Rneasy Mini kit (Qiagen, Mississauga, Ontario) and reverse transcription was performed on 1 µg total cellular RNA in a final volume of 20 µl using the QuantiTect RT kit. qPCR assays were designed with the Universal Probe Library from Roche (www.universalprobelibrary.com). Primers and probes for each gene are listed in Table S1 (xls spreadsheet file). Expression level for endogenous controls was determined using the following pre-validated Taqman assays (Applied Biosystems): TATA binding protein (TBP; assay number Hs00427620_m1) and ribosomal protein S9 (Hs00396989_m1). qPCR reactions for 384 well plate formats were performed using 2 µl of cDNA samples (5–25 ng), 5 µl of the Fast Universal qPCR MasterMix (Applied Biosystems), 2 µM of each primer and 1 µM of a UPL probe in a total volume of 10 µl. A control reaction without RT was performed on each run. An ABI PRISM 7900HT Sequence Detection System (Applied Biosystems) programmed with an initial step of 3 minutes at 95°C, followed by 45 cycles of 5 sec at 95°C and 30 sec at 60°C was used. All reactions were run in duplicate, averaged, and normalized to the averages of the housekeeping genes TBP and S9 to quantify the relative gene expression from that of the pre-treatment visit using the 2^−ΔΔCT^ method.

### Statistics

Statistical analysis was performed using the Vassar Web site (http://faculty.vassar.edu/lowry/VassarStats.html) and the GraphPad Prism software v5.0b statistical package. All tests were two-tailed, and *p* values <0.05 were considered significant.

## Results

### Intact TLR functions in MDCs enhance chronic hepatitis C treatment-induced viral clearance

**Figure 1:**
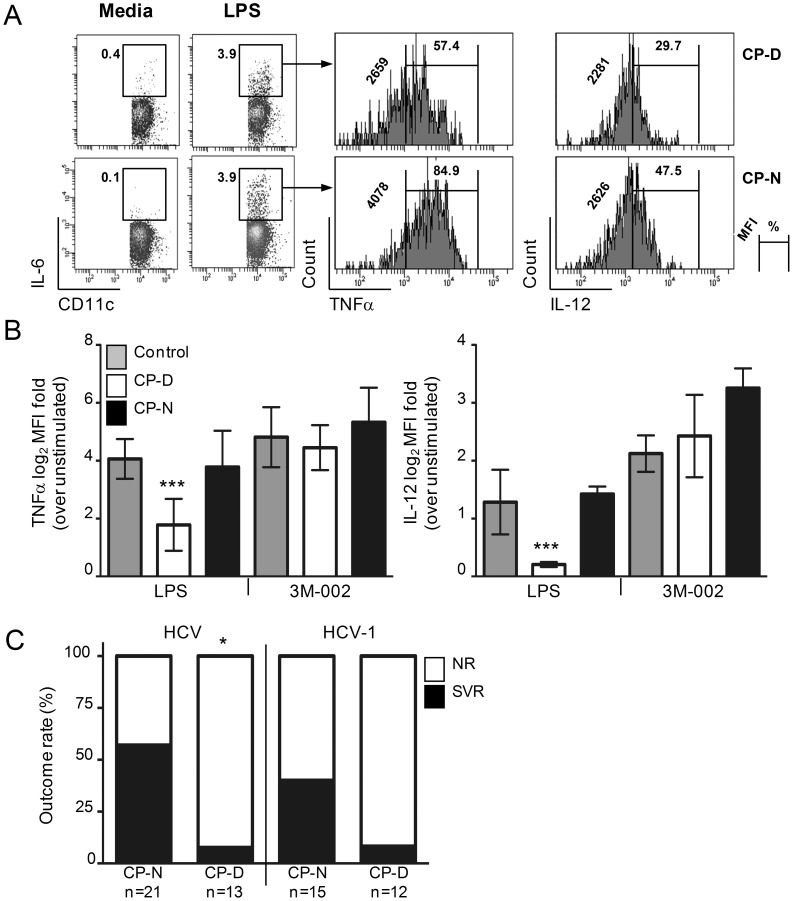
Lower rate of SVR in HCV chronic patients with impaired MDC TLR functionality. PBMCs were cultured in the presence of brefeldin A and TLR agonists for 6(A) Representative FACS plots of IL-6^+^ gated lineage^−^CD16^−^CD45^+^CD11c^+^MHC-II^br^ MDCs positive for TNF-α or IL-12 before treatment are shown following LPS or 3M-002 stimulation as stratified by CP cluster. Numbers on the left side of and above the bracketed lines indicate the geometric MFI and the percentages of cytokine–expressing MDCs in the designated area, respectively. (B) TNF-α and IL-12 expression (mean ± SD) as a log_2_ geometric MFI fold-induction above unstimulated control. Statistical comparisons between CP groups and aviremic control group were calculated by the Dunnett one-way ANOVA post test (***P<0.001). (C) MDC cytokine profile-stratified analysis of SVR and NR rates indicates that the difference between CP-N and CP-D patients irrespective of HCV genotypes is significant (n = 34 subjects; *P = 0.0048 by Fisher's exact two-tail test).

**Figure 2:**
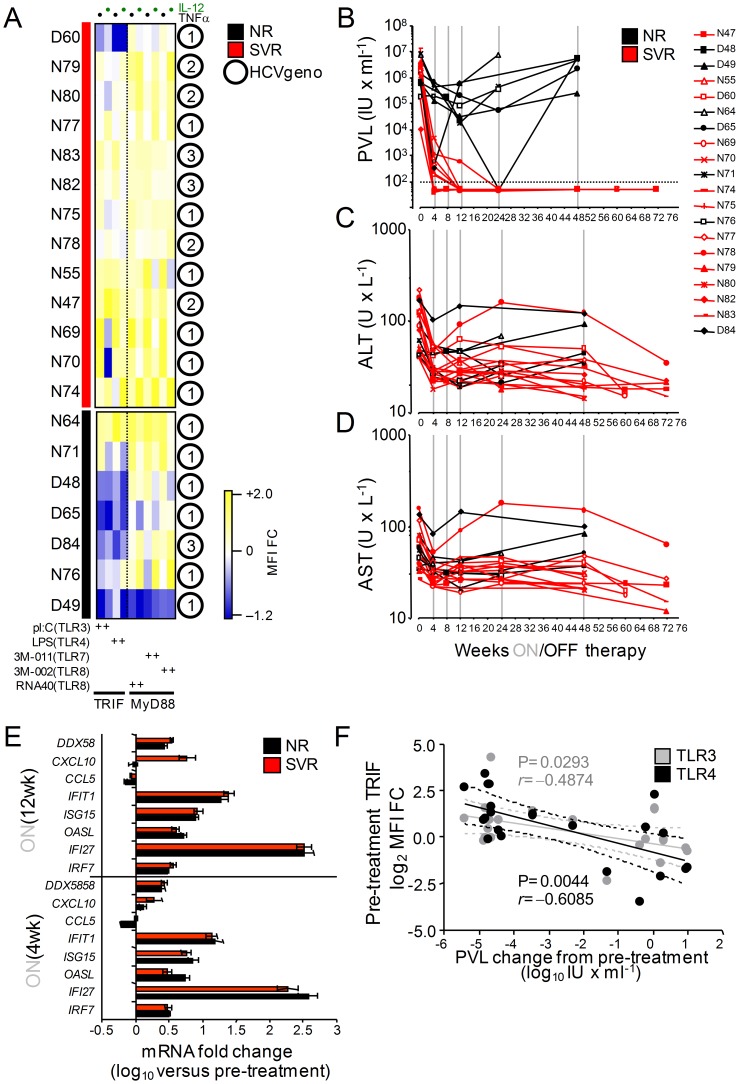
MDC TLR functionality is associated with the likelihood of achieving SVR following pegIFN and ribavirin treatment. (A) Heat maps of week 0 (pre-treatment) FACS measured IL-12 and TNFα (columns) protein expression profiles (CP) for lineage^−^CD16^−^CD45^+^CD11c^+^MHC-II^br^ MDCs activated with TLR agonists from viremics starting therapy and followed longitudinally (n = 20) as a log_2_ fold-change in MFI expression relative to TLR stimulated MDCs from a reference group of aviremic controls that cleared HCV after IFN therapy (n = 12) (yellow, higher than; blue, lower than; white, no change versus protein expression in aviremics). (B-D) Individual HCV plasma viral RNA loads (PVL, B), ALT (C) or AST (D) levels were determined before, during and after antiviral therapy. Red lines represent SVR; black lines NR. Vertical gray lines indicate period of antiviral treatment. (E) Relative levels of IRG mRNAs in PBMCS of NRs and SVRs at 4 and 12 weeks of treatment, normalized to pre-treatment levels, as determined by qPCR. Error bars represent mean ± SEM. (F) Correlation between MDC inhibition (sum of IL-12 and TNF-α TRIF-dependent TLR MFI fold-change, log_2_) prior to treatment initiation and the end-of-treatment changes of PVL from pre-treatment (n = 20).

Previous studies have postulated that the immune response of the host plays a role in clearance of viremia during IFN-based treatment of chronic HCV [20]. Support for this concept is, however, limited and less is known of the role played by DCs during treatment. In order to investigate the hypothesis that pre-treatment differences in HCV inhibition of DC pathogen sensing functions influences an individual's response to IFN therapy, we analyzed from a cohort of patients initiating pegIFN treatment 34 HCV-infected individuals that had completed the therapeutic regimen (Table 1) and in 20 of which an adequate number of longitudinal visits existed during and after cessation of therapy (Table 2). This study was designed and implemented from 2006–2010 and therefore does not include patients receiving DAAs and the analysis was not limited to genotype 1 patients. As previously reported [13], [16], a subgroup of HCV chronically infected patients (termed Cytokine-Profile Defective, CP-D, n = 13 out of 34) had MDCs that exhibited impaired production of IL-12 and TNFα production that was restricted to their TRIF-dependent TLR-sensing activities as opposed to a group of infected patients with intact MDC functions (CP-Normal, n = 21 out of 34; Table 1 and Figure 1A-B). Stratification of these 34 randomly selected treated patients according to treatment outcomes indicated that the TRIF-dependent TLR functional profile of DCs prior to therapy initiation provides an association with the virological outcome after pegIFN: 57% of CP-N analyzed (12 out of 21) had suppressed their plasma viremia after cessation of treatment as opposed to 8% of CP-D patients (1 out of 13; Figure 1C). Though a small sample size, the difference in SVR between CP-N and CP-D subjects was also apparent when only subjects infected with HCV genotypes 1 were examined (Figure 1C, P = 0.0914), suggesting that the difference in response is unlikely due to a biased distribution of difficult-to-treat HCV genotypes between the two DC cluster groups (Table 1). TLR-induced DC activation in response to a panel of TLR agonists, serum HCV RNA viral load (PVL) as well as aspartate transaminase (AST) and alanine transaminase (ALT) levels were determined at pre-treatment (week 0) and at 4, 8, 12, 24, and 48 weeks of pegIFN/ribavirin treatment in 20 patients followed longitudinally (Figure 2A-D). In the majority of patients with intact MDCs that were followed longitudinally (n = 15, denoted by an N before the ID number, Figure 2A), HCV replication at week 4 of therapy was considerably lower than that at pre-treatment (Figure 2B); the median reduction of PVL was −4.11 log_10_ IU/ml, which gradually reduced to <1.6 log_10_ IU/ml at 12 wks, and this decrease was paralleled by a decrease in serum AST and ALT levels (Figure 2C-D). The response of the five CP-Ds that were followed longitudinally was generally weaker and transient. At the end of treatment, HCV RNA was undetectable in 12 out of the 15 CP-N patients (80%) and AST/ALT levels normalized similarly in these 12 SVRs, whereas four of the five CP-Ds followed were nonresponders (NR) (Figure 2B-D). Levels of classical IFN-regulated genes (IRGs: IRF7, IFI27, OASL, ISG15, IFIT1, CCL5, CXCL10, DDX58) were strongly upregulated at week 4 and 12 of treatment in either group of subjects (Figure 2E), indicating that the difference between NR and SVR was at least independent of IFN action in the hematopoietic compartment. To determine whether HCV inhibition of MDCs or other initial biological parameter predicted the end-of-treatment PVL response, we performed a correlation analysis between all initial parameters that we measured and the 48-wk PVL change in these twenty patients. The pre-treatment TRIF-dependent TLR response potential of MDCs correlated negatively with the end-of-therapy decrease of PVL (r = −0.4874 [*P* = 0.0293] and r = −0.6085 [*P* = 0.0044], TLR3 and TLR4 respectively; Figure 2F). As expected, there was no correlation with MyD88-dependent TLR response potentials of MDCs (unpublished data), which are not inhibited by HCV (Figure 1B and 2A). Moreover, all other parameters measured (including pre-treatment PVL, age, gender, HCV-1 genotype, AST, ALT) did not correlate with end-of-therapy PVL change, further supporting a direct correlation between the pre-treatment functional status of MDCs in patients and their response to therapy. Altogether, these data suggest that lack of HCV inhibition of pathogen recognition by MDCs improves the host's ability to contain HCV infection once therapy is administered.

### Higher HCV-specific CD8+ T cells degranulation was associated with improved capacity to suppress viral replication during treatment

**Figure 3:**
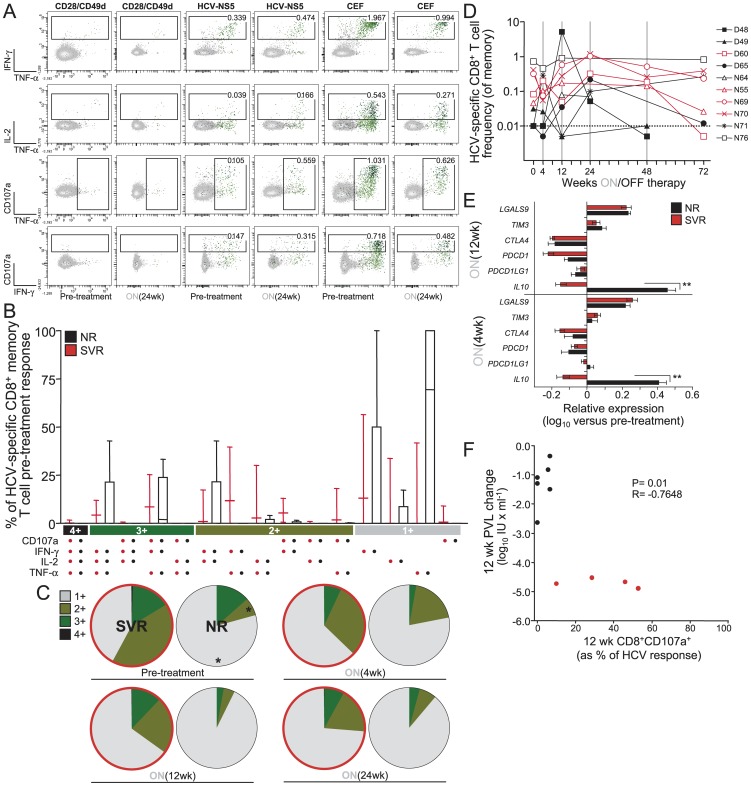
Differences in functional phenotype of the HCV-specific CD8 responses during therapy between SVRs and NRs. (A) Pre- and on-treatment FACS density plots in response to a 5.5 hour incubation with 2 µgml^−1^ of HCV NS5 peptide pools showing the frequencies of memory CD8^+^ T cells displaying the depicted combinations of CD107a mobilizing and intracellular production of IFNγ, IL-2, and TNF-α for patient P70. Gating was done on viable memory CD14^−^CD19^−^CD3^+^CD4^−^CD8^+^ cells that were not CD27^+^CD45RO^−^. Background activity against CD28/CD49d costimulation alone has been subtracted. (B) Flow cytometric analysis of polyfunctionality within total genotype 1 HCV-specific CD8 memory T-cells is shown at pre-treatment prior to start of therapy. The bar chart shows each of the 15 possible response profiles on the x-axis as the percentage of the total cytokine response on the y-axis. The filled bar represent the interquartile range and the line the median. (C) Summary of functional profile in SVR (red outline) and NR during treatment. The distinct cellular subsets shown in panel B were grouped by number of functions, so each section of the pie charts represent the mean proportion of HCV-specific CD8^+^ T cells grouped by the number of functions expressed independently of any particular function and matching the color code used in panel B. Statistically significant differences at pre-treatment between SVRs an NRs (*P<0.05, by Mann Whitney test) are indicated by the asterix. (D) The frequency of total HCV-specific CD8 memory T-cell responses at pre-treatment (week 0) and during treatment is shown in each individual as a percentage of their total memory CD8 T-cell population. (E) pegIFN-induced breakdown of the immunosuppressive environment is similar in nonresponders versus responders. Relative levels of immune suppressive genes in PBMCs of NRs (black) and SVRs (red), normalized to pretreatment levels, as determined by qPCR. Error bars represent mean ± SEM (Bonferoni's One-way Anova post-test, **P<0.001). (F) Relationship between the proportion of CD107a mobilization on HCV-specific CD8^+^ T cells at 12 weeks after treatment initiation and the 12-week treatment changes of PVL from pre-treatment (n = 10, analyzed using Pearson correlation). Each symbol corresponds to one subject: SVRs are in red, NRs are in black.

**Figure 4:**
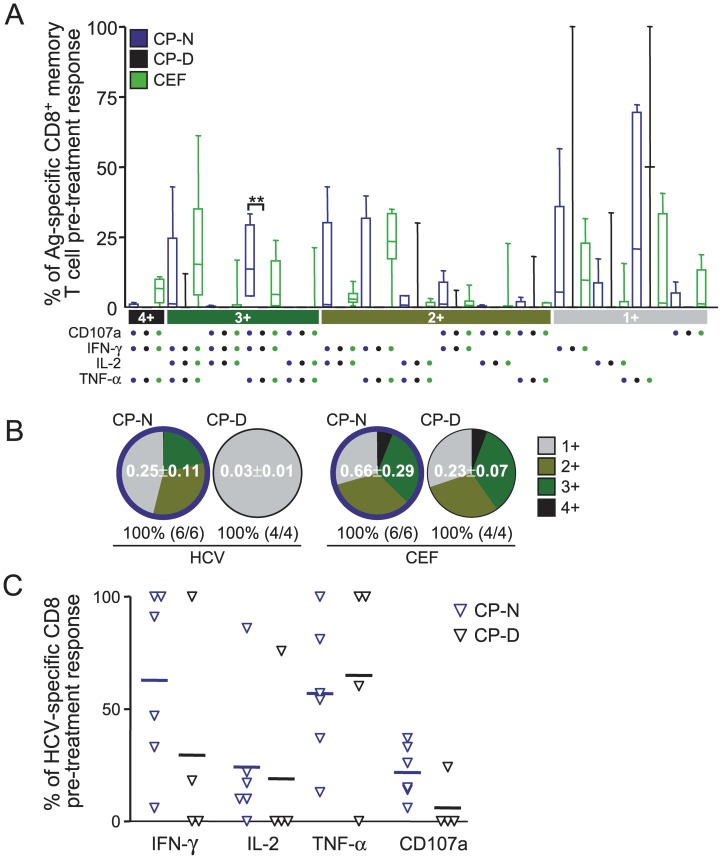
Enhanced HCV-specific CD8^+^ T cell polyfunctionality is restricted to patients that have intact MDCs. (A) Relative contribution to the total specific CD8^+^ response made by each function or function combination in pre-treatment samples stratified according to MDC TLR potential clustering. The filled bar represent the IQR, and the line the median. CD8 responses of patients with HCV inhibited MDCs (CP-D cluster) are shown in black versus those with intact MDCs (CP-N) in blue; responses to CMV, EBV, and influenza (CEF) peptides are shown in green for all chronically infected patients irrespective of CP clustering. Statistically significant differences (**P<0.001 by Kruskal-Wallis test) are indicated. (B) The pie charts show the HCV fractions according to the pie-slice colors shown at the bottom of the bar charts of panel A. Numbers in the pie are the mean total HCV-specific CD8 frequencies ± SE. For each individual, all positive peripheral blood responses to either Core, NS3, NS4 or NS5B pools (based on individual functional patterns) were summed to determine the total HCV-specific response within the memory peripheral blood T cell populations. (C) The proportion of HCV-specific CD8 memory T-cells producing IFNγ, IL-2, TNF-α and mobilizing CD107a (as determined by flow cytometric analysis after background subtraction) in CP clustered HCV genotype 1 patients at pre-treatment is shown. Lines are to the mean. The proportion of CD107a mobilizing cells was highest in the CP-N patients' responses (P = 0.06).

The more functions a CD8^+^ T cells can perform simultaneously is thought to be largely responsible for the control of viral infections (reviewed in [21]). In addition, TLR signaling deficiency has been reported recently to exacerbate T cell exhaustion in lifelong persistent viral infection [18]. Therefore, we hypothesized that MDCs displaying normal TLR functions might enhance HCV clearance rate under pegIFN therapy because these patients generally maintain improved HCV-specific CD8^+^ T cell polyfunctionality despite being chronically infected [13]. To evaluate this point, we used polychromatic flow cytometry and standard intracellular cytokine staining assays to measure cytokine production (IFN-γ, IL-2 and TNFα) and surface mobilization of CD107a (as a readout of cytotoxic degranulation) by CD8^+^ T cells following stimulation with overlapping peptide pools encompassing 4 proteins of genotype 1 HCV (Figure 3). The use of genotype 1 HCV peptides limited the T cell analysis to 14 of the 20 patients that were followed longitudinally, four of which were discarded from further analysis because the viability of some of their PBMC visit samples after thawing was below 95% as measured by trypan blue exclusion (see Table 2). Degranulating and cytokine-secreting HCV-specific CD8^+^ T cells could be measured at pre-treatment and during treatment (Figure 3A). We observed that HCV-specific CD8^+^ T cells from NRs exhibit reduced numbers of simultaneous functions prior to treatment initiation compared to SVRs (Figure 3B). In particular, at the start of therapy NRs had a significantly higher proportion of monofunctional cells (mean, 79.0%±14.5%) and significantly lower proportions of bifunctional cells (mean, 8.2%±17.2%) than SVR subjects (mean, 42.1%±20.9% [*P* = 0.016] and 41.4%±14.3% [*P* = 0.038], respectively; Figure 3C). Moreover, the proportion of these cells did not increase over time during treatment in either group (Figure 3C). Compared to the response frequency before treatment, a median 5.77 (3.55–17.74) fold increase in HCV-specific CD8 responses was observed during 24 weeks of treatment independently of treatment outcome (Figure 3D); a change associated with a reduction in the expression of exhaustion markers such as PD-1 (Figure 3E). Indeed, with the exclusion of IL-10 (*IL10*) (P<0.001), RNA expression levels of the negative immune regulators PD-1 (*PDCD1*), PD-L1 (*PDCD1LG1*), and CTLA-4 (*CTLA4*) were similarly decreased between NRs and SVRs during 4-12 weeks of treatment (Figure 3E), indicating that pegIFN is capable, albeit partly, to breakdown the extrinsic T cell immune suppressive environment regardless of the outcome of virus clearance. There was no significant change in the frequency or polyfunctionality of CEF-specific responses observed in the same individuals (Figure 3A). Moreover, we observed that HCV-specific CD8^+^ T-cells from SVRs consistently displayed an enhanced ability to degranulate *ex vivo* compared to NRs (Figure 3B), indicating that this function of HCV-specific CD8^+^ T cells could be relevant to virus control. Notably, there is an inverse relationship between the 12 week PVL change and HCV-specific CD107a surface mobilization on CD8^+^ T cells (Pearson R = −0.7648, P = 0.01; Figure 3F), suggesting that the capability of HCV-specific CD8^+^ T-cells to degranulate may represent a correlate of control not unlike what is reported for HIV infection [22]. At pre-treatment, enhanced polyfunctionality (Figure 4A-B) and the ability to mobilize CD107a (Figure 4C) was restricted to effector CD8^+^ T cells of CP-N patients that have MDCs with intact TLR sensing compared to those of CP-Ds and these properties of HCV-specific CD8^+^ T cells were generally not restored during therapy (Figure 3). Thus, intact innate immune functions of MDCs, which are associated with improved cytotoxic effector CD8^+^ T cell responses prior to therapy, likely help tip the balance to immune control and clearance of virus once immune suppressive signals are removed by antiviral treatment.

## Discussion

Multiple aspects of the HCV innate and adaptive immune response prior to therapy onset were characterized in a cohort of chronically infected subjects that completed the therapeutic regimen in order to delineate which of these functions associate with disease resolution upon treatment. We found that: (i) the extent to which HCV inhibits TRIF-dependent TLR response potentials of MDCs correlates with the end-of-therapy decrease of PVL (Figure 2), (ii) pre-treatment polyfunctional CD107a^+^ HCV-specific CD8^+^ T cells could be detected with higher incidence in SVRs and in patients that did not suffer from HCV inhibition of TLR sensing by MDCs (Figures 3 and 4); (iii) this higher proportion of HCV-specific CD8^+^ T cells able to degranulate was associated with virus control during treatment (Figure 3); and (iv) no improvement of T-cell polyfunctionality, as assessed by cytokine production and CD107a cytolytic activity, was induced by pegIFN during the first 24 weeks of therapy irrespective of treatment outcome (Figure 3). These findings indicate that, instead of being a marker of antiviral function, T-cell polyfunctionality is affected by viral inhibition of MDC functions. Exacerbated exhaustion of T-cell polyfunctionality during viremic chronic infection has recently been demonstrated to be a consequence of loss of TLR signals derived from MDCs [18]. Recently, the cytokine IL-12 was demonstrated to rescue the anti-viral functions of exhausted HBV-specific CD8^+^ T cells [23], further supporting the above concept. On the other hand and despite the small sample size, the observation that, out of all CD8^+^ T-cell functions studied, only the frequency of CD107a^+^ HCV-specific CD8^+^ T cells correlated with viral control (Figure 3) would suggest that not all functions exerted by a CD8^+^ polyfunctional T cell are equally relevant in mediating an anti-viral effect *in vivo*. The CTL functions reported in this study have been linked recently to virologic control in chronic HIV infection [24] and it will be interesting to see if these also holds true for HCV in larger scale studies.

MDC TLR functionality joins a considerable list of pre-treatment factors, that have been shown to associate with the probability of SVR, including HCV RNA concentration, HCV genotype, fibrosis stage and *IL28B* genotype [25]. However, individually none is sufficient to guarantee or to preclude the possibility of achieving an SVR. This study also contributes to our understanding of chronic HCV pathogenesis. We propose that when MDCs are inhibited by HCV, T cell dysfunctions are more severe and IFN therapy alone is unlikely to be highly beneficial because it disables rather than restores immune functions [26]–[28]. Numerous HCV induced immune defects are not reversed by IFNs in chronically infected patients, specifically the anti-viral functions of CD8^+^ T cells (this study and [29]). Moreover, IFNs in nonresponders appear to induce negative pathways like TAM receptors capable of broadly inhibiting TLR signaling [30] on innate immune cells (I.G.R.G., L.J. and D.L., unpublished data). Conversely, in a context of intact TLR sensing and secretion of IL-12 by MDCs, where T cell exhaustion is less severe as exemplified by preservation of CD107a mobilization, IFNs may prove more effective because they can remove the abundance of immunosuppressive signals (PD-1/PD-L1, CTLA-4 etc.), which would otherwise prevent expansion of pre-existing HCV-specific CD8^+^ T cells [13] capable of effectively controlling the infection [31], [32]. Thus, patients that harbour functional TRIF-dependent MDC activation pre-treatment responses are some of those who have a better chance of achieving on-treatment control of HCV replication because in these cases pegIFN is more likely to tip the balance towards sustained clearance of virus as they also happen to have improved CD107a^+^ CD8^+^ T cell polyfunctionality. Studies with nonhuman primates and humans support this concept: therapeutic vaccination, which recruits functional DCs into the APC pool, is more effective after antiviral therapy [33], [34]. The high efficacy of DAAs like NS3-4A protease inhibitors could potentially be due to their ability to rapidly restore TLR signaling of HCV inhibited MDCs as shown *in vitro* [13], in addition to their known direct antiviral effects [35]. Intriguingly, i.v. administration of the synthetic TLR7 agonist isatoribine reduced HCV plasma viremia in treatment-naïve chronically infected patients [36]. While therapy of HCV is rapidly evolving towards IFN-free regimen with combinations of different DAAs, current DAAs are generally administered in tandem with pegIFN [37]. Thus, the combination of DAAs with new adjunctive therapeutic strategies aimed at revitalizing DC functions that regulate the quality of the adaptive immune response during chronic HCV infection may help circumvent some of the issues relating to IFN use and assist in the development of an effective IFN-free regimens with high genetic barrier to resistance.

## Supporting Information

Table S1 Primers and probes for each gene listed in this study.
(XLS)

## References

[1] Mohd Hanafiah K, Groeger J, Flaxman AD, Wiersma ST (2013) Global epidemiology of hepatitis C virus infection: New estimates of age-specific antibody to HCV seroprevalence. Hepatology 57: 1333–1342. 23172780 10.1002/hep.26141

[2] Fried MW, Shiffman ML, Reddy KR, Smith C, Marinos G, et al (2002) Peginterferon alfa-2a plus ribavirin for chronic hepatitis C virus infection. N Engl J Med 347: 975–982. 12324553 10.1056/NEJMoa020047

[3] McHutchison JG, Everson GT, Gordon SC, Jacobson IM, Sulkowski M, et al (2009) Telaprevir with peginterferon and ribavirin for chronic HCV genotype 1 infection. N Engl J Med 360: 1827–1838. 19403902 10.1056/NEJMoa0806104

[4] Rong L, Dahari H, Ribeiro RM, Perelson AS (2010) Rapid emergence of protease inhibitor resistance in hepatitis C virus. Sci Transl Med 2: 30–32. 10.1126/scitranslmed.3000544 PMC3033690 20445200

[5] Welsch C, Jesudian A, Zeuzem S, Jacobson I (2012) New direct-acting antiviral agents for the treatment of hepatitis C virus infection and perspectives. Gut 61: i36–i46. 22504918 10.1136/gutjnl-2012-302144

[6] Lauer GM (2013) Immune responses to hepatitis C virus (HCV) infection and the prospects for an effective HCV vaccine or immunotherapies. Journal of Infectious Disease 207: S7–S12. 10.1093/infdis/jis762 23390305

[7] Ng CT, Sullivan BM, Oldstone MB (2011) The role of dendritic cells in viral persistence. Current Opinion in Virology 1: 160–166. 21909344 10.1016/j.coviro.2011.05.006 PMC3167161

[8] Lu W, Wu X, Lu Y, Guo W, Andrieu JM (2003) Therapeutic dendritic-cell vaccine for simian AIDS. Nature Medecine 9: 27–32. 10.1038/nm806 12496959

[9] Lu W, Arraes LC, Ferreira WT, Andrieu JM (2004) Therapeutic dendritic-cell vaccine for chronic HIV-1 infection. Nature Medecine 10: 1359–1365. 10.1038/nm1147 15568033

[10] Li S, Roberts S, Plebanski M, Gouillou M, Spelman T, et al (2012) Induction of multi-functional T cells in a phase I clinical trial of dendritic cell immunotherapy in hepatitis C virus infected individuals. PLoS One 7: e39368. 22905088 10.1371/journal.pone.0039368 PMC3419178

[11] Casazza JP, Bowman K, Adzaku S, Smith EC, Enama ME, et al.. (2013) Therapeutic Vaccination Expands and Improves the Function of the HIV-specific Memory T Cell Repertoire. Journal of Infectious Disease in press. 10.1093/infdis/jit098 PMC3654747 23482645

[12] Fuller MJ, Callendret B, Zhu B, Freeman GJ, Hasselschwert DL, et al (2013) Immunotherapy of chronic hepatitis C virus infection with antibodies against programmed cell death-1 (PD-1). Proceedings of the National Academy of Science USA 110: 15001–15006. 10.1073/pnas.1312772110 PMC3773803 23980172

[13] Rodrigue-Gervais IG, Rigsby H, Jouan L, Sauvé D, Sékaly RP, et al (2010) Dendritic cell inhibition is connected to exhaustion of CD8+ T cell polyfunctionality during chronic hepatitis C virus infection. Journal of Immunology 184: 3134–3144. 10.4049/jimmunol.0902522 20173023

[14] Wang N, Liang Y, Devaraj S, Wang J, Lemon S, et al (2009) Toll-like Receptor 3 Mediates Establishment of an Antiviral State Against Hepatitis C Virus in Hepatoma Cells. Journal of Virology 83: 9824–9834. 19625408 10.1128/JVI.01125-09 PMC2747996

[15] Dansako H, Yamane D, Welsch C, McGivern DR, Hu F, et al (2013) Class A Scavenger Receptor 1 (MSR1) Restricts Hepatitis C Virus Replication by Mediating Toll-like Receptor 3 Recognition of Viral RNAs Produced in Neighboring Cells. PLoS Pathogen 9: e1003345. 23717201 10.1371/journal.ppat.1003345 PMC3662657

[16] Rodrigue-Gervais IG, Jouan L, Beaulé G, Sauvé D, Bruneau J, et al (2007) Poly(I:C) and lipopolysaccharide innate sensing functions of circulating human myeloid dendritic cells are affected in vivo in hepatitis C virus-infected patients. Journal of Virology 81: 5537–5546. 17376921 10.1128/JVI.01741-06 PMC1900294

[17] Miyazaki M, Kanto T, Inoue M, Itose I, Miyatake H, et al (2008) Impaired cytokine response in myeloid dendritic cells in chronic hepatitis C virus infection regardless of enhanced expression of Toll-like receptors and retinoic acid inducible gene-I. Journal of Medical Virology 80: 980–988. 18428149 10.1002/jmv.21174

[18] Walsh KB, Teijaro JR, Zuniga EI, Welch MJ, Fremgen DM, et al (2012) Toll-like receptor 7 is required for effective adaptive immune responses that prevent persistent virus infection. Cell Host Microbe 11: 643–653. 22704624 10.1016/j.chom.2012.04.016 PMC3377981

[19] Ghany MG, Strader DB, Thomas DL, Seeff LB (2009) Diagnosis, management, and treatment of hepatitis C: an update. Hepatology 49: 1335–1374. 19330875 10.1002/hep.22759 PMC7477893

[20] Neumann AU, Lam NP, Dahari H, Gretch DR, Wiley TE, et al (1998) Hepatitis C viral dynamics in vivo and the antiviral efficacy of interferon-alpha therapy. Science 282: 103–107. 9756471 10.1126/science.282.5386.103

[21] Seder RA, Darrah PA, Roederer M (2008) T-cell quality in memory and protection: implications for vaccine design. Nature Review Immunology 8: 247–258. 10.1038/nri2274 18323851

[22] Hersperger AR, Migueles SA, Betts MR, Connors M (2011) Qualitative features of the HIV-specific CD8+ T-cell response associated with immunologic control. Current Opinion in HIV and AIDS 6: 169–173. 21399496 10.1097/COH.0b013e3283454c39 PMC4309378

[23] Schurich A, Pallett LJ, Lubowiecki M, Singh HD, Gill US, et al (2012) The third signal cytokine IL-12 rescues the anti-viral function of exhausted HBV-specific CD8 T cells. PLoS Pathogen 9: e1003208. 10.1371/journal.ppat.1003208 PMC3597507 23516358

[24] Hersperger AR, Pereyra F, Nason M, Demers K, Sheth P, et al (2010) Perforin expression directly ex vivo by HIV-specific CD8 T-cells is a correlate of HIV elite control. PLoS Pathogen 6: e1000917. 20523897 10.1371/journal.ppat.1000917 PMC2877741

[25] Thompson AJ (2012) Genetic factors and hepatitis C virus infection. Gastroenterology 142: 1335–1339. 22537440 10.1053/j.gastro.2012.01.046

[26] Teijaro JR, Ng C, Lee AM, Sullivan BM, Sheehan KC, et al (2013) Persistent LCMV infection is controlled by blockade of type I interferon signaling. Science 340: 207–211. 23580529 10.1126/science.1235214 PMC3640797

[27] Wilson EB, Yamada DH, Elsaesser H, Herskovitz J, Deng J, et al (2013) Blockade of chronic type I interferon signaling to control persistent LCMV infection. Science 340: 202–207. 23580528 10.1126/science.1235208 PMC3704950

[28] Tilton JC, Manion MM, Luskin MR, Johnson AJ, Patamawenu AA, et al (2008) Human immunodeficiency virus viremia induces plasmacytoid dendritic cell activation in vivo and diminished alpha interferon production in vitro. Journal of Virology 82: 3997–4006. 18256146 10.1128/JVI.01545-07 PMC2293017

[29] Abdel-Hakeem MS, Bédard N, Badr G, Ostrowski M, Sékaly RP, et al (2010) Comparison of immune restoration in early versus late alpha interferon therapy against hepatitis C virus. Journal of Virology 84: 10429–10435. 20668076 10.1128/JVI.01094-10 PMC2937796

[30] Rothlin CV, Ghosh S, Zuniga EI, Oldstone MB, Lemke G (2007) TAM receptors are pleiotropic inhibitors of the innate immune response. Cell 131: 1124–1136. 18083102 10.1016/j.cell.2007.10.034

[31] Wherry EJ (2011) T cell exhaustion. Nature Immunology 12: 492–499. 21739672 10.1038/ni.2035

[32] McMahan RH, Golden-Mason L, Nishimura MI, McMahon BJ, Kemper M, et al (2010) Tim-3 expression on PD-1+ HCV-specific human CTLs is associated with viral persistence, and its blockade restores hepatocyte-directed in vitro cytotoxicity. Journal of Clinical Investigation 120: 4546–4557. 21084749 10.1172/JCI43127 PMC2994339

[33] Hel Z, Venzon D, Poudyal M, Tsai WP, Giuliani L, et al (2000) Viremia control following antiretroviral treatment and therapeutic immunization during primary SIV251 infection of macaques. Nature Medicine 6: 1140–1146. 10.1038/80481 11017146

[34] Tryniszewska E, Nacsa J, Lewis MG, Silvera P, Montefiori D, et al (2002) Vaccination of macaques with long-standing SIVmac251 infection lowers the viral set point after cessation of antiretroviral therapy. Journal of Immunology 169: 5347–5357. 10.4049/jimmunol.169.9.5347 12391256

[35] Lamarre D, Anderson PC, Bailey M, Beaulieu P, Bolger G, et al (2003) An NS3 protease inhibitor with antiviral effects in humans infected with hepatitis C virus. Nature 426: 186–189. 14578911 10.1038/nature02099

[36] Horsmans Y, Berg T, Desager JP, Mueller T, Schott E, et al (2005) Isatoribine, an agonist of TLR7, reduces plasma virus concentration in chronic hepatitis C infection. Hepatology 42: 724–731. 16116638 10.1002/hep.20839

[37] Liang TJ, Ghany MG (2013) Current and future therapies for hepatitis C virus infection. New England Journal of Medicine 368: 1907–1917. 23675659 10.1056/NEJMra1213651 PMC3893124

